# Symptomatic Bilateral Sacroiliitis in a Patient with Juvenile Systemic Lupus Erythematosus: A Rare Association

**DOI:** 10.31138/mjr.34.1.105

**Published:** 2023-03-31

**Authors:** Vikas Gupta, Devinderpal Singh Dhanota

**Affiliations:** 1 Department of Rheumatology & Immunology, Dayanand Medical College & Hospital, Ludhiana, Punjab, India,; 2 Department of Radiodiagnosis, Dayanand Medical College & Hospital, Ludhiana, Punjab, India

**Keywords:** juvenile systemic lupus erythematosus, sacroiliitis, juvenile spondyloarthropathy, inflammatory back pain

## INTRODUCTION

Musculoskeletal manifestations are seen in about 70% to 95% of Systemic Lupus Erythematosus (SLE) patients during the disease course, with joint pain as the initial symptom in about 50% of patients.^1,2^ Joints affected most commonly in Lupus arthritis are hand joints, including metacarpophalangeal, proximal interphalangeal and distal interphalangeal joints, and knees. Shoulders, ankles, and elbows can also be affected though less commonly.^3^ However, sacroiliac joint involvement is not typical of SLE and is limited to case reports. We describe here an adolescent male with SLE who developed inflammatory back pain two years after the diagnosis of SLE and was found to have bilateral sacroiliitis on Magnetic Resonance imaging (MRI). To the best of our knowledge, this is the first case of juvenile SLE having bilateral symmetric sacroiliitis. Previously, one patient with juvenile SLE having unilateral sacroiliitis has been described who was later diagnosed as juvenile spondyloarthropathy after ruling out sacroiliac joint infection.^4^

## CASE REPORT

A 13-year-old boy diagnosed with Systemic Lupus Erythematosus (SLE) for two years, on low dose steroids, Mycophenolate Mofetil and hydroxychloroquine, presented with intermittent low back pain for two months. The course of SLE over the last two years included cutaneous (malar rash), haematological involvement (thrombocytopenia) and renal involvement (proteinuria of 1.2 g/d). Renal biopsy was not done, however, since he had thrombocytopenia. At the time of diagnosis, he had positive anti-nuclear antibody (ANA) by immunofluorescence (3+ homogenous at 1:80 dilution), strongly positive anti-dsDNA and low C3 and C4 complement levels. Lupus anticoagulant was transiently positive (positive at diagnosis but negative when repeated 3 months later). Anticardiolipin IgM and IgG antibodies, and anti-beta-2-glycoprotein 1 IgM and IgG antibodies were negative.

**Figure 1.**
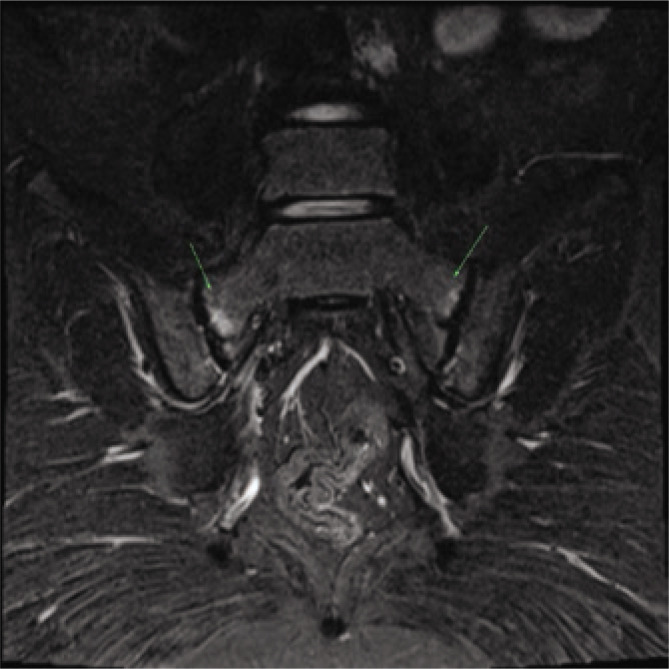
Coronal STIR images showing bilateral subchondral bone marrow edema and articular surface irregularity of bilateral sacroiliac joints.

**Figure 2.**
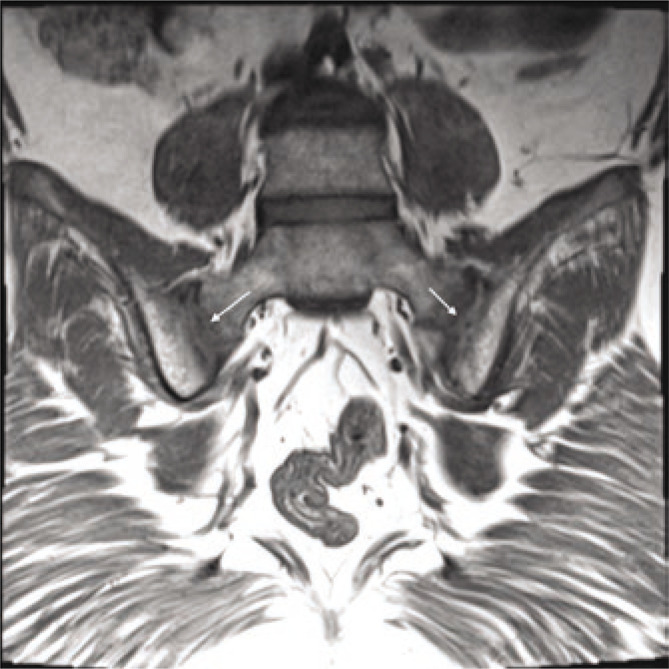
Coronal T1 weighted images showing articular surface erosions of bilateral sacroiliac joints.

**Figure 3.**
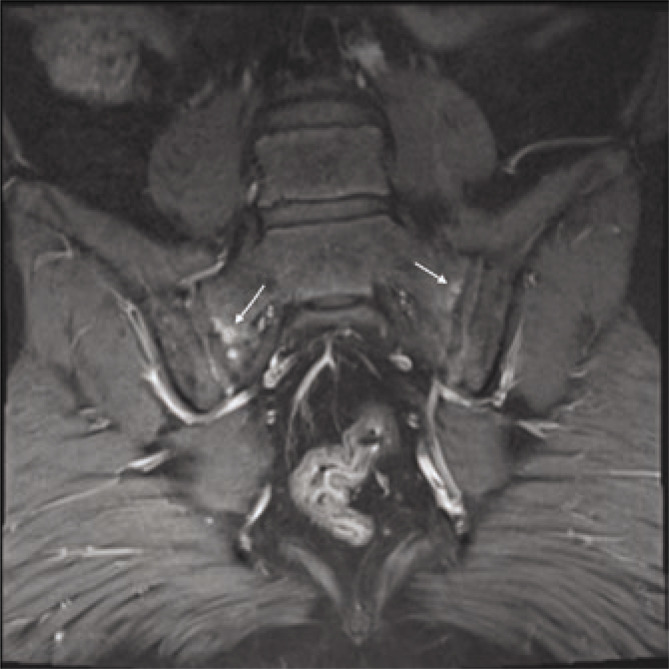
Gadolinium-enhanced coronal T1 weighted images showing subchondral enhancement in bilateral sacroiliac joints.

Two years after diagnosis of SLE, he presented with pain and swelling of bilateral metacarpophalangeal joints which improved with increase in dose of steroids. Methotrexate (MTX) was added in view of arthritis of small joints of hands since the patient’s parents declined Rituximab. A few weeks later, while his hand joint arthritis had resolved, he developed intermittent low back pain. His back pain was inflammatory in nature, occurring more on waking up in the morning and associated with early morning stiffness of about half an hour. Pain would recur after every 3–4 days and was occasionally severe and sometimes associated with fever ranging from 100 to 101 degrees Fahrenheit. There was no history suggestive of enthesitis. There was excellent response to non-steroidal anti-inflammatory drugs (NSAIDs) which the patient used to take on as required basis. C-reactive protein (CRP) value increased from 12 to 76 mg/L over a period of two months. He was evaluated for low back pain and fever. Magnetic resonance imaging (MRI) of sacroiliac joints and spine was done which revealed bilateral symmetric active sacroiliitis (**Figures 1–3**). Human Leukocyte Antigen (HLA) B27 was negative by polymerase chain reaction (PCR). There was no evidence of any infective pathology on the MRI spine. Other work up for fever did not show any evidence of infection. Procalcitonin was normal. Complement C3 and C4 levels were within normal range. Other SpA manifestations, including uveitis, enthesitis, or a family history of HLA B27-related disease or psoriasis/psoriatic arthritis were absent. Hence, the patient was started on full dose NSAIDs (Naproxen 500 mg twice daily, weight 67 kg). Patient showed excellent response to treatment and his low back pain and fever disappeared completely within the next few days. His MTX dose was stepped up to 20 mg/week in view of predominant musculoskeletal manifestations and Mycophenolate dose reduced. After three weeks of continuous NSAIDs and MTX 20 mg/week, his CRP fell from 76 mg/L to 20 mg/L. His NSAIDs requirement came down significantly and these were gradually stopped. He has been followed for six months now and has tolerated the combination of MTX and low-dose MMF well. Currently he is in clinical remission on 20 mg weekly Methotrexate, 1 g daily Mycophenolate, 200 mg daily Hydroxychloroquine and 2.5 mg daily prednisolone.

## DISCUSSION

Inflammatory back pain (IBP) and sacroiliitis are mainly features of spondyloarthropathies and are not typically seen in SLE. We here report a patient of juvenile SLE who developed inflammatory back pain and on evaluation was found to have acute bilateral sacroiliitis on MRI. Sacroiliitis has been rarely reported in SLE. To the best of our knowledge, though ten cases of coexistent SLE and SpA have been reported in adults, there is only a single case report of coexistent juvenile SLE and juvenile SpA.^4^ In this previous report, a 16-year-old girl with SLE had unilateral sacroiliitis on MRI and a diagnosis of juvenile SpA was made after ruling out infective sacroiliitis.^4^ In contrast, our patient, who developed inflammatory back pain two years after the diagnosis of SLE, had acute bilateral sacroiliitis on MRI. The imaging features, including subchondral bone marrow oedema, subchondral enhancement, and erosions in bilateral sacroiliac joints, were similar to those usually seen in axial spondyloarthritis (AxSpA). However, no synovial enhancement within the joint to suggest synovitis was seen in our patient.

The prevalence of sacroiliitis in SLE is unclear as the data on the same is scarce.^3^ Two prospective studies from Mexico and Turkey have evaluated the presence of IBP and sacroiliitis in adult SLE patients. While 10% to 16% of adult SLE patients reported IBP in these studies, grade 2 or higher radiographic sacroiliitis (unilateral or bilateral) was found in 2% to 10% of patients.^5,6^ Sacroiliitis in SLE has been reported to be generally mild and not linked to HLA-B27.^6^ Our patient also had negative HLA-B27 and his back pain responded well to NSAIDs. In case the sacroiliitis in our patient gets active and is not controlled with NSAIDs and conventional DMARDs, we would consider off-label use of JAK inhibitors (JAKi). Currently, TNF inhibitors (TNFi), IL-17 inhibitors (IL-17i) and JAKi are recommended for the management of active sacroiliitis in AxSpA that is not controlled on NSAIDs.^7,8^ However, these drugs are not recommended for the management of SLE and have only been used in exceptional circumstances as suggested from a few published case reports and case series.^9^ Among these agents, TNFi infliximab has been used successfully in five patients with lupus arthritis who achieved remission but relapsed 2–3 months after the last dose.^10^ However, increase in autoantibody titres (anti-dsDNA and anti-cardiolipin) and development of lupus-like symptoms seen with infliximab, deters most clinicians from using TNFi in SLE.^10^ Secukinumab, an IL-17i, has been used successfully in an SLE patient with refractory lupus nephritis, however, IL-17i have been reported to induce lupus erythematosus in a few patients.^11–13^ JAKi (tofacitinib and baricitinib), have shown improvement in arthritis and skin rash in SLE patients without any major safety concerns.^14,15^ Currently, these are being evaluated in multiple clinical trials.^16^ Hence, the authors believe JAKi to be the most promising agents among the various available biologic and targeted synthetic DMARDs for the treatment of active sacroiliitis in SLE refractory to NSAIDs and conventional DMARDs.

In conclusion, we report a rare case of juvenile SLE with sacroiliac joint involvement. Our case re-emphasises the need to keep a high index of suspicion for sacroiliitis in a patient of SLE presenting with inflammatory back pain, even in the adolescent age group.
